# Evaluating pharmacist involvement in HIV outpatient clinics: can medication histories, drug interaction checks and adherence assessments add benefit?

**DOI:** 10.1186/1758-2652-13-S4-P115

**Published:** 2010-11-08

**Authors:** K Seden, DJ Back, SH Khoo

**Affiliations:** 1 NIHR Biomedical Research Centre, Royal Liverpool & Broadgreen University Hospitals Trust, Liverpool, UK; 2 University of Liverpool, Department of Pharmacology and Therapeutics, Liverpool, UK

## Purpose of study

HIV patients often take complicated regimens with high propensity for drug-drug interactions (DDIs). Physician awareness has been found to be low, and recognition relies on a comprehensive and current medication history. Patients may receive treatment from various sources such as other hospital departments, their GP, over the counter from a pharmacy or via the internet. Patients may also use herbal medicines, vitamins or supplements, some of which have potential to affect antiretroviral (ARV) therapy. Recreational drugs may also impact ARV treatment, either by affecting drug levels or adherence. This study aims to assess whether detailed medication history taking, screening for DDIs and adherence checks by a pharmacist are beneficial in HIV outpatient clinics.

## Methods

Consecutive patients taking ARVs were seen by a pharmacist prior to outpatient medical review. Patients were asked for a detailed medication history including ARVs, hospital prescribed medication, medication prescribed in primary care, over the counter medication, herbal medicines, vitamins, supplements and recreational drugs. Adherence to ARVs was assessed using a modified MASRI scale. The medication list was screened for DDIs, and a personalised interaction printout from www.hiv-druginteractions.org placed in the clinical notes. Physicians were asked to respond via a brief questionnaire whether the information told them something they did not know, and if they changed management of the patient as a result.

## Summary of results

Of 90 patients, 20(22%) were taking a prescribed medication which was not previously recorded, 10(11%) had a discontinued medication recorded in their notes and 8(8.8%) were taking a different dose than that recorded. 43(48%) patients had one or more DDI, with a total of 70 DDIs identified. Estimated adherence ranged from 60-100%. 57 physician responses were obtained as shown in Table 1:

**Table 1:** Summary of Physician Responses

	Told me something I did not know (%)	Changed management of the patient (%)
Medication History	10 (18)	2 (4)

DDI Check	18 (32)	6 (11)

Adherence Check	22 (39)	2 (4)

## Conclusions

Detailed medication history taking by pharmacists has an application in HIV outpatient clinics. Routine screening of HIV patients' full medication lists for DDIs can facilitate recognition of interactions which may otherwise not be identified and managed. These data suggest that pharmacist led consultations incorporated into HIV outpatient clinics can add benefit.

